# Application of symptom-based mind mapping combined with PBL teaching method in emergency trauma standardized resident training in MDT model

**DOI:** 10.1097/MD.0000000000030822

**Published:** 2022-09-23

**Authors:** Zhou-Wei Xu, Na-Na Liu, Jian-Lin Zhang, Xue-Sheng Wu, Jia Chen, Jia-Wei Chang, Bai-Cheng Ding, Yu-Nuo Wu, Jia-Peng Wang, Wei-Dong Chen, Xing-Yu Wang

**Affiliations:** a Department of Emergency Surgery, The First Affiliated Hospital of Anhui Medical University, Hefei, Anhui, P.R. China; b Institute of Clinical Pharmacology, Anhui Medical University, Key Laboratory of Anti-inflammatory and Immune Medicine, Ministry of Education, Anhui Collaborative Innovation Center of Anti-inflammatory and Immune Medicine, Hefei, Anhui, P.R. China; c Department of Clinical Medical, the First Clinical Medical College of Anhui Medical University, Hefei, Anhui, P.R. China.

**Keywords:** emergency trauma, MDT model, mind mapping, PBL teaching, standardized residency training

## Abstract

Explore the feasibility and effectiveness of accepting mind mapping combined with problem-based learning (PBL) teaching method in the standardized training of emergency surgery residents in the multi-disciplinary team (MDT) model of emergency trauma. Eighty-nine doctors under training who rotated in the Department of Emergency Surgery of the First Affiliated Hospital of Anhui Medical University from January 2021 to January 2022 were selected as the study subjects, and randomly divided into a group receiving mind mapping combined with PBL teaching and a group receiving traditional lecture-based learning teaching. Mini-clinical evaluation exercise (Mini-CEX), direct observation of procedural skills (DOPS), teaching adherence, and satisfaction assessments were completed at the time of discharge from the department. There were no significant differences between the observation and control group trainees in terms of gender, age, education, and entry grades. Both groups of doctors were better able to participate in their respective teaching modes and made significant progress. The participants in the observation group had significantly higher Mini-CEX, DOPS, and teaching satisfaction scores than the control group (*P *< .05). Under the MDT model of emergency trauma, the combination of mind mapping and PBL teaching can improve the comprehensive clinical ability of the trainees more than participating in the traditional lecture-based learning teaching, which is worth promoting and implementing in the clinical standardized training.

## 1. Introduction

Standardized residency training is the first mandatory stage of post-graduation education for clinical medical students. By receiving systematic and standardized training of comprehensive clinical competence as a resident in a nationally recognized hospital with training qualifications, the aim is to develop the 4 major job competencies of resident’s professional ethics, professional and technical skills, teamwork and communication skills, and teaching and research skills. This type of training is currently the mainstream way of training physicians around the world.^[1–3]^ The First Affiliated Hospital of Anhui Medical University is a national training base for emergency surgery. Its training and teaching work has now become another important work content after clinical medical treatment, medical research, and undergraduate teaching. Therefore, combining the common emergency trauma cases in the department, adopting scientific and effective teaching methods, and developing a reasonable and fair assessment system is of outstanding significance to improve the emergency diagnosis and treatment level of the standardized clinical residents.

Emergency trauma patients, especially those with combined multiple injuries, are characterized by complex injuries, rapid changes in condition, many comorbidities, and easy misdiagnosis and underdiagnosis. In such cases, the multi-disciplinary team (MDT) mode is usually used to formulate targeted treatment plans by experts from different specialties and perform early controlled surgical interventions when necessary, which can significantly improve the patient’s outcome and prognosis.^[4,5]^ This approach is now widely used in our emergency surgery department. By participating in the MDT process of emergency trauma, it can establish a standardized and individualized treatment concept for the trainees and then cultivate their all-around and multi-dimensional treatment thinking ability. On this basis, further optimization of the teaching program will have a multiplier effect on the cultivation of qualified emergency medicine trainees. In the process of teaching practice at home and abroad, it is found that the problem-based learning (PBL) can effectively develop students’ ability to identify, analyze and solve problems.^[6–8]^ It is characterized by student-centered, case-oriented, and problem-based teaching and learning tasks in the form of group discussions. However, PBL teaching alone cannot make students form a systematic understanding of the learning content. In contrast, mind maps use a combination of text and graphics to make abstract problems concrete and simplify complex problems by linking fragmented knowledge together, so they are a good supplement to PBL teaching mode.^[9]^

We selected 89 standardized clinical residents who rotated in the Department of Emergency Surgery of the First Affiliated Hospital of Anhui Medical University from January 2021 to January 2022 as the study population. We introduced the symptom-based mind maps combined with PBL teaching method into the emergency trauma training teaching in MDT mode and applied the mini-clinical evaluation exercise (Mini-CEX) and direct observation of procedural skills (DOPS) assessments as formative evaluation tools, and achieved satisfactory teaching results, which can provide a basis for promoting the clinical training teaching method of emergency surgery in the future, which is reported as follows.

## 2. Materials and Methods

### 2.1. Research subjects

Among the 89 doctors in the department of emergency surgery who participated in the teaching study, 54 were male, and 35 were female. The age ranged from 22 to 29 years old, with an average of (24.21 ± 1.77) years old. Forty-seven had a bachelor’s degree, 32 had a master’s degree, and 10 had a doctoral degree. The randomized data table method was applied to divide the study population into observation and control groups, and theoretical performance was assessed after admission to the department. There were no significant differences between the members of the 2 groups in terms of general information (Table 1). Inclusion criteria of the study subjects: Obtained a clinical practitioner’s license; Rotation cycle in the department of emergency surgery for >1 month; Did not receive prior training related to mind mapping and PBL teaching; Informed and agreed to this study. Exclusion criteria: Failed to pass the entrance examination; Dropped out of the rotation cycle in the department of emergency surgery for <1 month or halfway; Could not cooperate to complete the clinical teaching training content; Participating in other studies.

**Table 1:** Comparison of the members of the 2 groups in general information.

Group	Number of cases	Gender		Age	Education			Entry grades
		Male	Female	(yr, χ±*s*)	Bachelor	Master	Doctor	(score, χ±*s*)
Observation group	45	28	17	23.91 ± 1.83	25	14	6	80.26 ± 5.48
Control group	44	26	18	24.52 ± 1.68	22	18	4	78.59 ± 4.61
*χ*^2^/*t*		0.096		1.342	1.080			0.937
*P*		.762		.184	.583			.405

### 2.2. Faculty team

The Department of Emergency Medicine selects the instructors of the training physicians, and the specific requirements include a master’s degree or above, intermediate title or above. Working years ≥5 years. Pass the teacher training course examination or obtain the teacher qualification certificate. Clinical experience and qualification to participate in emergency trauma MDT. Complete Mind Maps, PBL, Mini-CEX, and DOPS training and pass the examination. The MDT is composed of experts from the departments of emergency surgery, anesthesiology, critical care medicine, orthopedics, general surgery, thoracic surgery, and neurosurgery. In addition to having rich clinical knowledge and business ability in their specialty, members are also able to provide appropriate teaching guidance to the trainees.

### 2.3. Research methods

The 89 doctors who participated in the teaching study were randomly divided into an observation group and a control group, in which the participants in the observation group received the mind mapping combined with PBL teaching in the emergency trauma MDT model, while the participants in the control group only participated in the traditional lecture-based learning (LBL) teaching in the emergency trauma MDT model. Each cohort of trainees had a 4-week learning and assessment period in the emergency surgery department. The Mini-CEX and DOPS assessments were conducted in the 1st and 4th weeks after admission to the department, respectively. The assessment was scored by 3 non-instructors with intermediate or higher titles, and the average was taken as the final score. The differences in scores between the members of 2 groups while before and after enrollment were analyzed, and finally, the participants were surveyed for teaching adherence and satisfaction. The above research contents have been approved by the Clinical Medical Research Ethics Committee of the First Affiliated Hospital of Anhui Medical University (Quick-PJ2022-04-42).

### 2.4. Emergency trauma MDT teaching model

The MDT model is now widely used in the context of medical specialty integration and exchange. Through multidisciplinary coordination, the injury condition of emergency trauma patients is evaluated to provide appropriate treatment plans. If necessary, emergency surgery is carried out by the corresponding department according to the severity of each organ damage. In the MDT mode, the success rate of emergency trauma treatment can be significantly improved by reevaluating the injury condition and determining the subsequent treatment or surgery plan after the patient’s vital signs have stabilized. We involve the trainees in the MDT discussion and medical treatment process, during which the teachers of each specialty guide the participants to think about the problems that may arise, aiming to cultivate the trainees’ ability of comprehensive and integrated analysis of emergency trauma.

### 2.5. Mind mapping teaching based on symptoms

The observation group trainees applied X-Mind software to draw mind maps of emergency trauma under the guidance of their instructors, focusing on the principles of mapping: Place the injured organ in the center with symptoms as the core theme. The secondary issues are arranged in a radial pattern, concise, and clearly layered. Use different colors or pictures to express the content as much as possible to enhance the visual impact. The diagram should reflect the holistic thinking of trauma and be easy to understand and remember.^[10]^ Based on the selected emergency trauma PBL cases combined with problem-oriented, the training participants extracted keywords to construct the basic framework of mind maps. By using lines, characters, and pictures, they can link the relevant knowledge points from the center to the periphery. At the same time, the contents of the mind maps are expanded with the help of textbooks, clinical guidelines, and domestic and international literature. Trainees are encouraged to discuss during the process of making symptom-based mind maps, actively ask questions to the instructor to avoid discussion bias, and enhance their sense of teamwork.

### 2.6. PBL teaching

The instructor applied the PBL teaching content and mind maps to the teaching of the observation group. The case materials were distributed after the teaching of basic theory and clinical training of emergency trauma in small groups. At the same time, 4~5 questions were set from the diagnosis, treatment, surgical management, and doctor-patient relationship of the disease. Each group coordinates with each other to consult tools and literature, and after independent study, they compile an outline of the presentation and draw mind maps. Participants will first discuss the issues involved in the case within the group. Then the group representative will present the case analysis conclusion according to the content of the mind maps. And finally, the instructor will make a summary evaluation after the group discussion. The instructor can organize the trainees to select some typical emergency trauma cases for simulation training. The group members will play different roles to deepen their understanding of the knowledge.

### 2.7. Teaching effectiveness evaluation

The Mini-CEX and DOPS were used as formative evaluation tools for teaching effectiveness, with the assessment period being the first and fourth week of admission, respectively. The Mini-CEX focused on assessing the trainees’ receiving and handling skills, while the DOPS focused on assessing the trainees’ clinical operation skills.^[11]^ The Mini-CEX evaluation form we developed according to the characteristics of emergency trauma includes 7 aspects such as interrogation ability, communication skills, professionalism, physical examination, clinical judgment, operational ability, and overall evaluation, etc.^[12,13]^ Each item adopts a stanine scale in 3-level, with 1~3 being substandard, 4~6 being qualified, and 7~9 being excellent. The DOPS evaluation form judged the trainees’ performance in the clinical practice activities of trauma. The assessment is drawn from routine surgical operations such as debridement and suturing, suture removal, abdominal puncture, catheterization, and gastric tube insertion.^[14]^ The criterion is based on an internationally accepted 6-point scale in 4-level, with 1 to 2 being below the expected standard, 3 being close to the expected standard, 4 being at the expected standard, and 5 to 6 being above the expected standard. The degree of cooperation of the trainees with the respective teaching mode is classified into full compliance, basic compliance, partial compliance, and noncompliance according to the level. The results are used as important indicators for evaluating the final teaching effect. The teaching satisfaction assessment is conducted by means of a questionnaire out of the department, which mainly includes stimulating learning interest, deepening knowledge understanding, improving clinical thinking and recognizing the teaching mode, etc. Participants are rated from 0 to 10 points according to their actual experience.

### 2.8. Statistical processing

All data were statistically analyzed using SPSS 23.0 software, and the measurement data were expressed as χ ± *s*, while *t* test was used for comparison between groups. Count data were expressed as the number of cases or percentages, while *χ*^2^ test was used for comparison between groups, and *P *< .05 indicated that the difference was statistically significant.

## 3. Results

### 3.1. Mind maps of acute trauma in the MDT model

**Figure 1.**
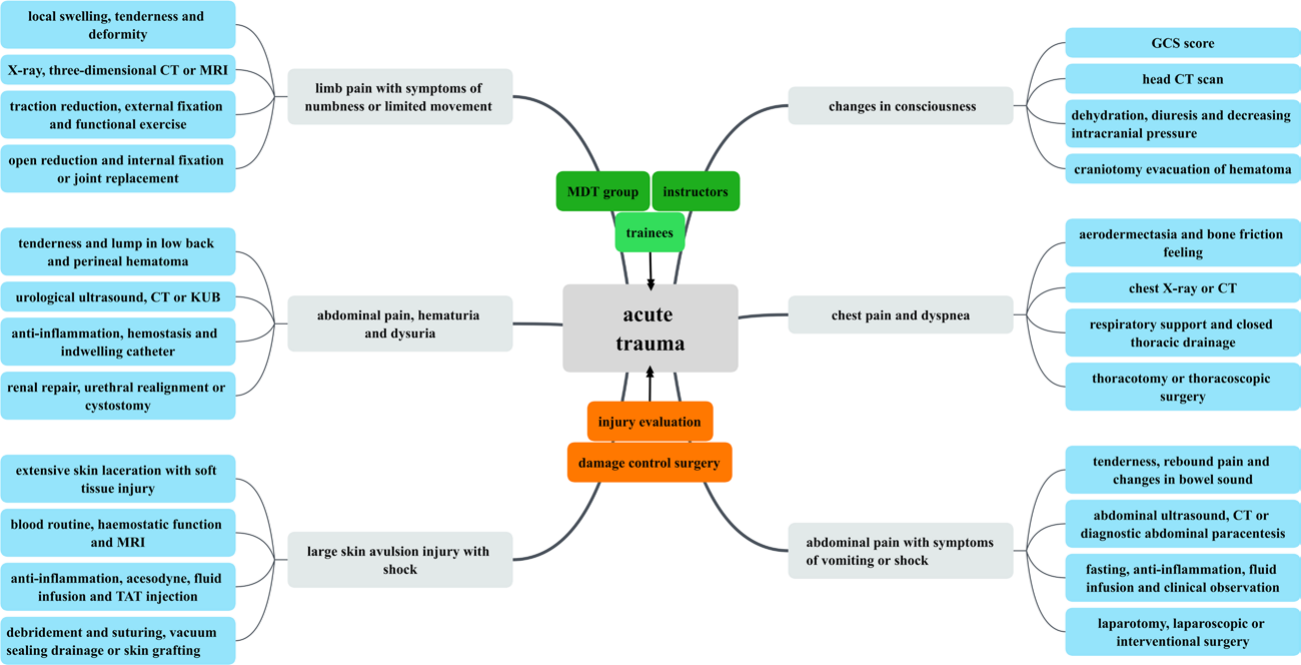
Symptom-based mind mapping of acute trauma in the MDT model. MDT = multi-disciplinary team.

The observation group of trainees attending the basic trauma theory training in the department was able to participate in the MDT treatment and teaching process actively. This has a certain depth in understanding of the assessment of emergency trauma, consultation and treatment process, treatment principles, surgical selection, and postoperative rehabilitation, and has the ability to complete the reception and initial treatment of patients with multiple and compound injuries under the guidance of teachers in MDT. Most of the trainees in the observation group could correctly grasp the principles of drawing mind maps and the application of X-Mind software, review the literature through teamwork to make symptom-based emergency trauma mind maps and complete the summarization of the whole consultation process of trauma patients in the guide map (Fig. 1).

### 3.2. The integration of PBL teaching and mind maps

**Figure 2.**
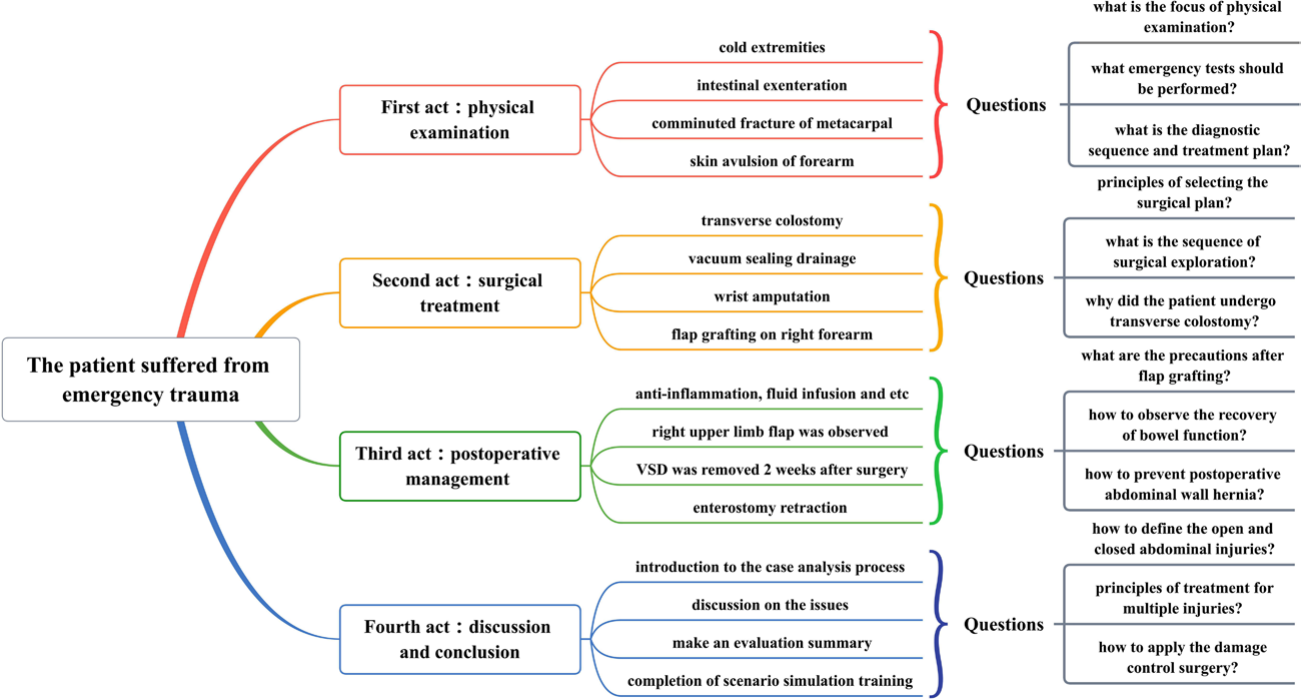
The integration of PBL teaching and mind mapping in the case of acute trauma. PBL = problem-based learning.

Under the guidance of the instructors, the observation group of trainees showed high enthusiasm for the PBL teaching format. They were able to take the initiative to identify, analyze and solve problems based on the distributed case materials. Group members developed appropriate treatment plans for emergency trauma patients by reviewing literature and participating in discussions, and correctly played the roles chosen in the situational simulation exercises. The participants of the observation group introduced the method of drawing mind maps, which had been mastered in the previous period, into the PBL case analysis, so that the symptoms, auxiliary examinations, diagnosis, and treatment of the disease were presented in an orderly manner as holistic thinking, making the clinical decision-making process easy to understand and remember, and we used the case of emergency trauma in Figure 2 to elaborate.

The basic situation of the case: The patient was a male, 36 years old, who accidentally caught his right upper limb and abdominal clothes in a machine at work, resulting in crushing crush injury of the right hand, large skin avulsion injury of the abdomen and open abdominal injury, and was transferred to a higher level hospital after dressing the trauma at the local hospital. The patient was admitted with an indifferent expression, wet and cold extremities, total defect of the median abdomen with intestinal exenteration, severe crush injury of the right forearm, and comminuted fracture of the metacarpal. Blood pressure was 106/52 mm Hg, emergency blood count: WBC 16.4 × 10^9^/L, NEU% 90.5%, RBC 3.02 × 10^9^/L, Hb 87g/L; electrocardiogram showed sinus tachycardia. Questions in the first act: What parts of the physical examination should be focused on? What are the indifference of expression and cold extremities suggest? What emergency tests should be performed on this patient? What is the diagnostic sequence and treatment plan based on the patient’s condition?

An abdominal exploration in MDT mode revealed a ruptured transverse colon and mesenteric contusion, with no significant abdominal other organ abnormalities, and a post-repair transverse colostomy was performed. Some of the skin and soft tissues of the middle abdomen were missing. However, it was still possible to close the abdominal cavity with sutures, and the incision was drained with vacuum sealing drainage. The crushed fracture of the right hand could not be repaired, so a wrist amputation was performed, and a full-thickness skin flap graft was made on the skin defect of the right forearm. Questions in the second act: Principles of selecting the surgical plan? What is the sequence of surgical exploration? Why did the patient undergo external transverse colon surgery?

The patient was treated with symptomatic treatment such as anti-inflammatory, nutritional rehydration, etc. After the intestinal function was restored, the gastric tube was removed, and the diet was gradually resumed. The right upper limb flap was observed for abnormal blood flow 1 week after surgery during the hospitalization. At 2 weeks postoperatively, the abdominal incision was confirmed to be well-healed, and then the vacuum sealing drainage was removed. The external transverse colon was placed back into the abdominal cavity under local anesthesia at 4 weeks postoperatively. Questions in the third act: How to observe the recovery of bowel function? What are the precautions after flap grafting? What should be done if the patient develops a postoperative abdominal wall hernia?

In the discussion session, the trainees introduced the whole case analysis process in the form of mind maps and chose the corresponding roles to conduct scenario simulation exercises, in which the summary points of concern include: What is the definition of open and closed abdominal injuries? What are the principles of treatment for multiple injuries? What is the clinical application of damage control surgery?

### 3.3. Comparison of the performance of 2 groups of participants before and after teaching

**Figure 3.**
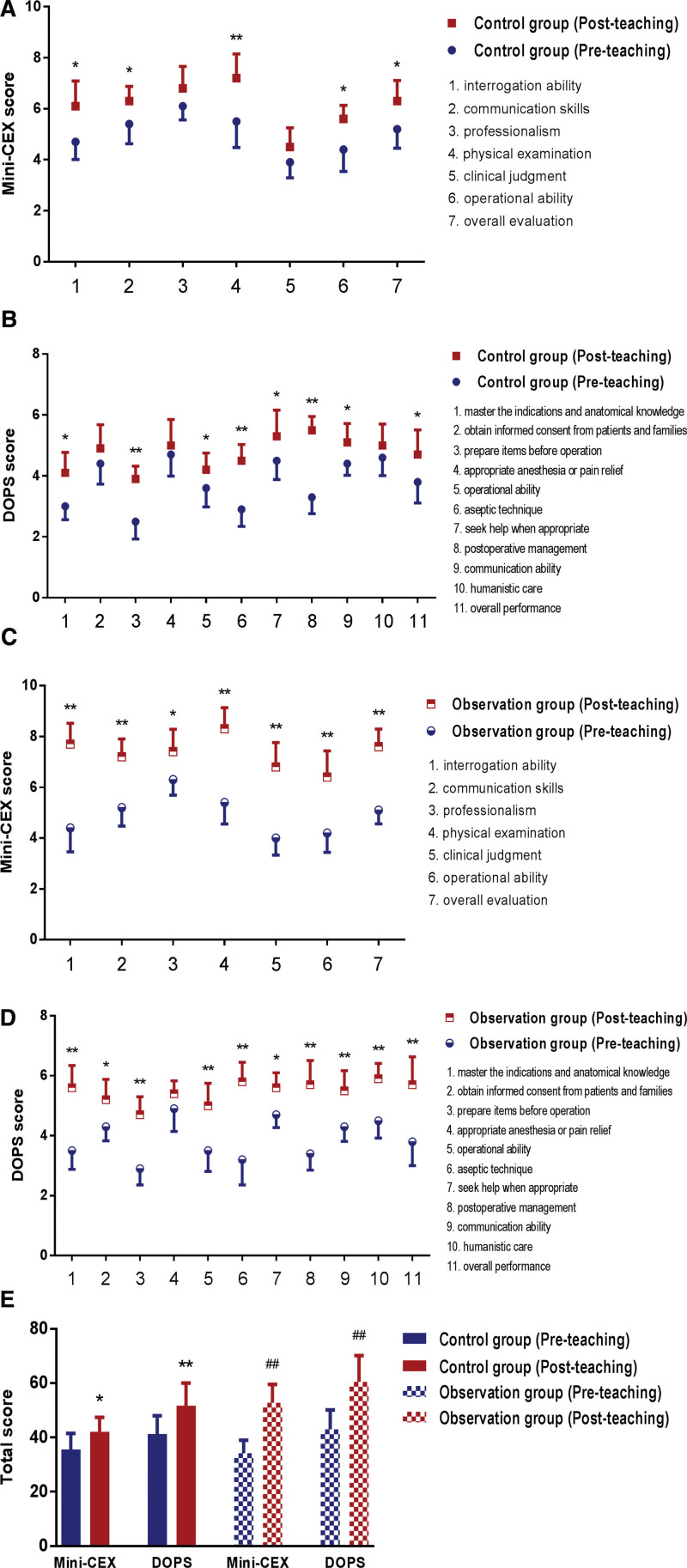
Comparison of the members of the 2 groups in performance before and after teaching. (A and B) The individual scores of Mini-CEX and DOPS of the control group before and after admission to the department. * *P* < .05, ** *P* < .01 VS the control group (pre-teaching). (C and D) The individual scores of Mini-CEX and DOPS of the observation group before and after admission to the department. * *P* < .05, ** *P* < .01 VS the observation group (pre-teaching). (E) The overall scores of Mini-CEX and DOPS of the 2 groups before and after admission to the department. * *P* < .05, ** *P* < .01 VS the control group (pre-teaching); ## *P* < .01 VS the observation group (pre-teaching). DOPS = direct observation of procedural skills, Mini-CEX = mini-clinical evaluation exercise.

The Mini-CEX and DOPS assessments were administered before and after the residents from observation and control groups participated in the teaching study. By analyzing the changes in the performance of the members of the 2 groups before and after admission to the department, the progress of their receiving and handling abilities and clinical operation skills during the study in the department of emergency surgery was assessed. The results of the study confirmed that the post-teaching Mini-CEX and DOPS scores of the 2 groups of trainees were significantly higher than the pre-teaching scores. There were significant differences in their overall scores and most individual scores, suggesting that within the emergency trauma MDT model, both the acceptance of mind maps combined with PBL teaching and the traditional LBL teaching can improve the overall level of the trainees, as detailed in Figure 3.

### 3.4. Comparison of teaching effectiveness between the 2 groups of participants

**Figure 4.**
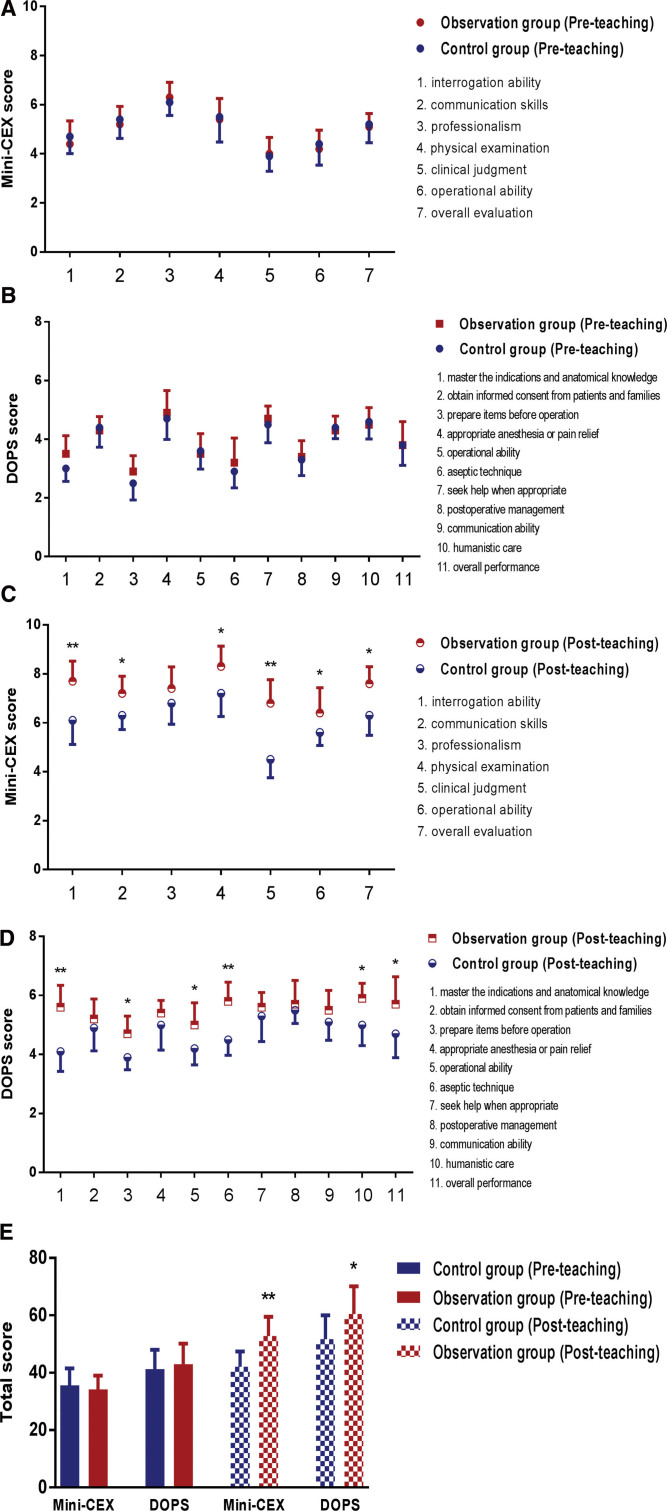
Comparison of teaching effectiveness between the members of the 2 groups. (A and B) The individual scores of Mini-CEX and DOPS between the 2 groups before admission to the department. (C and D) The individual scores of Mini-CEX and DOPS between the 2 groups after admission to the department. (E) The overall scores of Mini-CEX and DOPS between the 2 groups after admission to the department. * *P* < .05, ** *P* < .01 VS the control group (post-teaching). DOPS = direct observation of procedural skills, Mini-CEX = mini-clinical evaluation exercise.

The residents from observation and control groups were evaluated before and after the teaching study in terms of receiving and handling abilities and clinical operation skills, and the differences in their teaching effects under different training modes were assessed by analyzing the Mini-CEX and DOPS scores before and after the members of the 2 groups were admitted to the department. The results of the study confirmed that there was no significant difference between the Mini-CEX and DOPS scores of the 2 groups of trainees before teaching. Still, after teaching, the observation group had significantly higher overall scores and most individual scores than the control group, suggesting that within the emergency trauma MDT model, receiving mind maps combined with PBL teaching could improve the overall level of the trainees compared with the traditional LBL teaching, as detailed in Figure 4.

### 3.5. Evaluation of teaching adherence of 2 groups of participants

The adherence of the both groups of trainees to participate in different teaching modes has an important influence on the final teaching effect. Therefore, they were evaluated comprehensively in terms of the initiative of learning, the standardization of practice, and the cooperation of assessment, respectively. According to the level of compliance, they were classified as fully compliant, basically compliant, partially compliant, and non-compliant. The study identified full compliance and basic compliance as better compliance. The results confirmed that both the observation and control groups of participants showed better compliance in their respective teaching activities, with no significant differences between them (Table 2).

**Table 2:** Comparison of the members of the 2 groups in teaching adherence (n [%]).

Group	Number of cases	Full compliance	Basic compliance	Partial compliance	Noncompliance	Adherence
Observation group	45	24	12	7	2	36 (80.0%)
Control group	44	30	9	4	1	39 (88.6%)
*χ* ^2^						1.253
*P*						.278

### 3.6. Teaching satisfaction evaluation of 2 groups of participants

The teaching satisfaction evaluation of the both groups of trainees before they left the department was carried out using our homemade questionnaire, which included stimulating learning interest, deepening knowledge understanding, improving clinical thinking, enhancing teamwork, recognizing the teaching mode and overall satisfaction, and all the members who participated in the study completed the questionnaire. The summary results of the questionnaire showed that the observation group had significantly higher overall scores and all individual scores than the control group, suggesting that the participants were more satisfied with receiving mind maps combined with PBL teaching in the emergency trauma MDT model (Table 3).

**Table 3:** Comparison of the members of the 2 groups in teaching satisfaction.

Group	Number of cases	Stimulating learning interest	Deepening knowledge understanding	Improving clinical thinking	Enhancing teamwork	Recognizing teaching mode	Overall satisfaction	Overall scores
Observation group	45	8.53 ± 1.06	8.84 ± 1.13	8.09 ± 1.35	9.13 ± 1.04	9.02 ± 1.18	8.78 ± 1.41	52.39 ± 7.36
Control group	44	7.82 ± 1.29	8.25 ± 1.22	7.30 ± 0.93	7.61 ± 1.47	8.41 ± 1.02	7.73 ± 1.28	47.12 ± 6.84
*t*		2.875	2.386	3.231	5.658	2.625	3.672	4.014
*P*		.005	.019	.002	<.001	.011	<.001	<.001

## 4. Discussion

In the 21st century, medical science is rapidly developing and facing serious challenges. Medical educators are proposing a variety of teaching reforms for medical students at all stages of theoretical teaching, clinical practice, and residency training in order to produce qualified clinical medical personnel for society.^[15–17]^ Emergency medicine is a very distinctive emerging clinical medicine specialty whose level reflects to a certain extent of the comprehensive medical level of a hospital or even a country.^[18]^ As an inseparable part of emergency medicine, emergency surgery has become an independent discipline with rapid development. The training of qualified emergency surgeons is an important part of our clinician training.^[19]^ Therefore, we have participated in a number of clinical teaching training activities organized by society and the hospital, hoping to improve the effect of training and teaching in emergency surgery through the adoption of appropriate teaching methods. By combining the characteristics of the common emergency trauma patients in the department, we decided to carry out the teaching of emergency trauma MDT in the mode of receiving mind maps combined with PBL teaching and traditional LBL teaching, so as to provide a theoretical basis for further promoting the experience of training and teaching in the future.

The intervention of emergency trauma patients in MDT mode and damage control surgery when necessary can significantly reduce the mortality rate and improve the prognosis of patients.^[20]^ As a basic treatment model for modern diseases, MDT is conducive to the development of a multidisciplinary mindset, and trainees can integrate theoretical medical knowledge and clinical specialty practice by participating in the treatment process of emergency trauma patients.^[21,22]^ In our teaching study, we found that both groups of trainees had significantly improved their assessment scores after participating in the MDT model of emergency trauma teaching, suggesting that this method is practical and feasible to be applied to the training teaching. The trainees in the observation group introduced PBL teaching on the basis of MDT through discovering, analyzing, and solving problems in typical cases, which prompted standardized clinical residents to deepen their understanding of theoretical knowledge in teaching practice. It aims to stimulate learning interest, improve self-learning ability, cultivate clinical thinking and strengthen teamwork spirit. As a good complement to PBL teaching, the mind map is a note-taking method created by Tony Buzan, a famous British psychologist, to record the thinking process through lines, images, and markers in a radioactive way.^[23,24]^ Emergency trauma patients mostly have obvious clinical symptoms, so the symptom-based mind maps can adopt the technique of combining graphics and text to connect the topics of disease occurrence, progression, treatment, and prognosis at all levels with hierarchical diagrams, so as to improve the ability of participants to integrate clinical information and deal with clinical problems, thus effectively promoting their enthusiasm and efficiency of independent learning.

The development of a reasonable and fair assessment system in standardized training can help to evaluate the teaching effect objectively. Mini-CEX and DOPS can assess the trainees’ performance and give feedback at the same time, prompting them to recognize the shortcomings in learning and make targeted improvements.^[25]^ In this study, we found that the post-teaching Mini-CEX and DOPS assessment scores of both groups were significantly higher than the pre-teaching scores. The post-teaching assessment scores of the observation group were higher compared with the control group, suggesting that within the emergency trauma MDT model, receiving mind maps combined with PBL teaching can improve the comprehensive clinical competence of the trainees more than participating in the traditional LBL teaching. If the participants are not able to cooperate with the teaching activities or see them as a burden, it will directly affect the final learning performance.^[26]^ In our practice, we found that both groups of trainees were able to adapt well to their respective teaching methods. All participating doctors completed the satisfaction questionnaire before they left the department. The results confirmed that the trainees had higher recognition and interest in learning under the MDT model of emergency trauma with the combination of mind maps and PBL teaching, so this teaching model has better prospects for clinical promotion.

## 5. Conclusion

In summary, the clinical teaching of resident doctors is an important part of carrying out teaching reform, and the selection of appropriate teaching methods is the key to cultivating high-quality medical talents. This study found that the acceptance of mind maps combined with PBL teaching in the emergency trauma MDT model as a new teaching tool for emergency surgery trainees can both motivate participants to learn and improve clinical practice in a multi-dimensional way. Its good feedback was obtained in the teaching evaluation system, and it is worth being promoted and implemented in the standardized residency training of hospitals around the world.

## Acknowledgments

The authors are thankful to all staff members of Department of Emergency Surgery, The First Affiliated Hospital of Anhui Medical University, for their help in instructing the work.

## Author contributions

Zhou-Wei Xu contributed to the conception of the study and wrote the manuscript; Jian-Lin Zhang, Xue-Sheng Wu, and Jia Chen participated in the teaching study as the instructors; Jia-Wei Chang and Bai-Cheng Ding performed the teaching effectiveness evaluation; Na-Na Liu, Yu-Nuo Wu, and Jia-Peng Wang helped perform the data collection and analysis; Wei-Dong Chen and Xing-Yu Wang contributed to the research program and constructive discussions.

**Data curation:** Jia-Wei Chang.

**Formal analysis:** Jia-Wei Chang.

**Funding acquisition:** Zhou-Wei Xu.

**Investigation:** Na-Na Liu, Jia-Wei Chang.

**Project administration:** Jian-Lin Zhang, Xue-Sheng Wu, Jia Chen, Wei-Dong Chen.

**Resources:** Bai-Cheng Ding, Yu-Nuo Wu, Jia-Peng Wang.

**Supervision:** Wei-Dong Chen, Xing-Yu Wang.

**Validation:** Jia Chen, Bai-Cheng Ding.

**Writing – original draft:** Zhou-Wei Xu, Yu-Nuo Wu, Jia-Peng Wang.

**Writing – review & editing:** Xing-Yu Wang.
